# Preparation of *Auricularia auricula*-Derived Immune Modulators and Alleviation of Cyclophosphamide-Induced Immune Suppression and Intestinal Microbiota Dysbiosis in Mice

**DOI:** 10.3390/life15081236

**Published:** 2025-08-04

**Authors:** Ming Zhao, Huiyan Huang, Bowen Li, Yu Pan, Chuankai Wang, Wanjia Du, Wenliang Wang, Yansheng Wang, Xue Mao, Xianghui Kong

**Affiliations:** 1 Institute of Microbiology, Heilongjiang Academy of Sciences, Harbin 150000, China; zm1209_qqrr@163.com (M.Z.); hhy244025132@163.com (H.H.); l398867672@163.com (B.L.); panyu198701@163.com (Y.P.); 15765208277@163.com (C.W.); duwj010708@163.com (W.D.); 2 School of Nursing, Heilongjiang Agricultural Reclamation Vocational College, Harbin 150025, China; 3 Shandong Academy of Agricultural Sciences, Jinan 250100, China; cywwl@163.com (W.W.); sdnky_wys@163.com (Y.W.); 4 College of Agriculture and Biology, Liaocheng University, Liaocheng 252059, China

**Keywords:** *Auricularia auricula*, polypeptide, polysaccharide, immunomodulation, intestinal microbiota

## Abstract

With the acceleration of the pace of life, increased stress levels, and changes in lifestyle factors such as diet and exercise, the incidence of diseases such as cancer and immunodeficiency has been on the rise, which is closely associated with the impaired antioxidant capacity of the body. Polypeptides and polysaccharides derived from edible fungi demonstrate significant strong antioxidant activity and immunomodulatory effects. *Auricularia auricula*, the second most cultivated mushroom in China, is not only nutritionally rich but also offers considerable health benefits. In particular, its polysaccharides have been widely recognized for their immunomodulatory activities, while its abundant protein content holds great promise as a raw material for developing immunomodulatory peptides. To meet the demand for high-value utilization of *Auricularia auricula* resources, this study developed a key technology for the stepwise extraction of polypeptides (AAPP1) and polysaccharides (AAPS3) using a composite enzymatic hydrolysis process. Their antioxidant and immunomodulatory effects were assessed using cyclophosphamide (CTX)-induced immune-suppressed mice. The results showed that both AAPP1 and AAPS3 significantly reversed CTX-induced decreases in thymus and spleen indices (*p* < 0.05); upregulated serum levels of cytokines (e.g., IL-4, TNF-α) and immunoglobulins (e.g., IgA, IgG); enhanced the activities of hepatic antioxidant enzymes SOD and CAT (*p* < 0.05); and reduced the content of MDA, a marker of oxidative damage. Intestinal microbiota analysis revealed that these compounds restored CTX-induced reductions in microbial α-diversity, increased the abundance of beneficial bacteria (*Paramuribaculum*, *Prevotella*; *p* < 0.05), decreased the proportion of pro-inflammatory *Duncaniella*, and reshaped the balance of the Bacteroidota/Firmicutes phyla. This study represents the first instance of synergistic extraction of polypeptides and polysaccharides from *Auricularia auricula* using a single process. It demonstrates their immune-enhancing effects through multiple mechanisms, including “antioxidation-immune organ repair-intestinal microbiota regulation.” The findings offer a theoretical and technical foundation for the deep processing of *Auricularia auricula* and the development of functional foods.

## 1. Introduction

Since the 21st century, the acceleration of living rhythms, increased stress, and changes in lifestyle factors such as diet and exercise have contributed to a rising prevalence of diseases such as cancer and tumors [1]. One of the reasons for this phenomenon is the low, declining, or disordered immune function caused by the imbalance between the body’s antioxidant capacity and free radical metabolism, leading to the occurrence of diseases such as cancer and immune deficiency as well as the process of aging [2]. Therefore, people have sought safe and non-toxic natural antioxidants to enhance immunity, improve sub-healthy constitutions, and prevent the onset of tumors and cancers. Given the urgent needs of Chinese residents for immune enhancement and nutritional health, as well as the widespread demand for methods of preventing cancer and immune deficiency diseases, bolstering immunity has emerged as an effective strategy for self-protection and the maintenance of physical health. The market demand for immune-regulating products is substantial, and the prospects for developing such products are promising.

When studying immune enhancers, it is necessary to evaluate them using immune suppression models. In research reports, one of the most common model establishment methods is the use of cyclophosphamide (CTX) as an immunosuppressant. CTX is widely used clinically in anticancer therapy and immunosuppression [3,4]; however, its use also brings serious hazards. During CTX therapy, significant induction of in vivo oxidative stress occurs, leading to various types of tissue damage. Metabolites of CTX, such as acrolein and phosphoramide mustard, trigger excessive production of reactive oxygen species (ROS) [5]. This excessive ROS generation further results in lipid peroxidation, protein oxidation, and DNA damage. The overproduction of ROS disrupts intracellular redox homeostasis, activates inflammatory and apoptotic signaling pathways, and ultimately leads to tissue dysfunction [6]. CTX has a strong immunosuppressive effect, which leads to impaired immune system function and makes the body vulnerable to infections. CTX also has significant adverse effects on the hematopoietic system, potentially causing anemia, leukopenia, and thrombocytopenia, further increasing the risk of bleeding and infection [7]. Additionally, CTX can cause adverse digestive system reactions such as nausea, vomiting, and loss of appetite, affecting the quality of patients’ life. Long-term use of CTX may lead to damage to organs such as the liver and kidneys and even increase the risk of other malignant tumors [8].

Levamisole hydrochloride is a broad-spectrum immunomodulator that exerts a bidirectional regulatory effect on the body’s immune function. In cases of immune dysfunction or suppression, it can restore the impaired functions of macrophages and T cells to normal levels, thereby enhancing the body’s immune response capacity [9]. This includes promoting the production of lymphokines by T cells and strengthening the phagocytic function of phagocytes, among other effects [10]. Its mechanism of action and efficacy are relatively clear and stable, making it suitable as a positive control in immunological experiments to verify the validity of the experimental system.

Edible fungi are rich in high-quality proteins, carbohydrates, and other nutrients. Their protein content, accounting for 19% to 40% of dry weight, is higher than most vegetables [11]. They can serve as raw material sources for high-quality bioactive peptides, which can be prepared via enzymatic hydrolysis. Polypeptides and polysaccharides derived from edible fungi exhibit significant physiological activities, such as antioxidant, immunomodulatory, and antibacterial activities [12,13]. The *Pleurocinus ostreatus* polysaccharide can increase the body weight, spleen, and thymus indices of immune-suppressed mice; upregulate the levels of mouse immunoglobulins (IgM, IgG, and IgA) and cytokines, including tumor necrosis factor-α (TNF-α), interleukin-2 (IL-2), interleukin-6 (IL-6), and interferon-γ (IFN-γ); and reverse intestinal microbiota dysregulation in mice by increasing the abundance of *Muribaculaceae*, *Lactobacillaceae*, *Blautia*, and *Ligilactobacillus* [14]. The *Pleurotus abiticola* polysaccharide can alleviate CTX-induced histopathological damage and increase the expression of CD4, CD8, and CD56 in mouse splenocytes [15]. The protein from *Pleurotus eryngii* mushroom feet can significantly enhance the activities of intracellular antioxidant enzymes such as superoxide dismutase (SOD), glutathione peroxidase (GSH-Px), and catalase (CAT) and reduce intracellular levels of malondialdehyde (MDA) and ROS [16].

*Auricularia auricula* is the second most widely consumed edible fungus in China. The country possesses abundant auricula resources, and its production ranks first globally [17]. The polysaccharide content in *Auricularia auricula* can exceed 60%, including soluble polysaccharides (soluble dietary fiber) and insoluble polysaccharides (insoluble dietary fiber) [17]. The insoluble dietary fiber in *Auricularia auricula* includes chitin, cellulose, lignin, etc. [18], which can be enzymatically hydrolyzed into soluble dietary fiber. However, China’s *Auricularia auricula* industry suffers from weak deep-processing capabilities and low product added value. When extracting active components from *Auricularia auricula* fruiting bodies, only a portion of soluble polysaccharides can be extracted [19]. A large amount of insoluble dietary fiber remains in the post-extraction residues, which not only reduces the added value of *Auricularia auricula* processing but also causes environmental pollution. The sustainable development of the *Auricularia auricula* industry requires driving by deep-processed products, and product processing needs core technological breakthroughs.

This study uses the same batch of *Auricularia auricula* as raw material. Based on the principle of comprehensive utilization of active components, key technologies for preparing polypeptides and polysaccharides with high antioxidant activity were established through stepwise sequential extraction combined with a complex enzymatic hydrolysis process. This approach achieved the co-production of both components from a single batch of raw material within a single production process. Additionally, the in vivo immune-enhancing activity was verified using CTX-induced immune-suppressed mice, providing a research foundation for in-depth studies on the immune-enhancing effects of bioactive polypeptides and polysaccharides from *Auricularia auricula* and the development of high-value-added functional deep-processed products.

## 2. Materials and Methods

### 2.1. Chemicals and Reagents

*Auricularia auricula* was purchased from the edible fungus base in Heilongjiang province (Heilongjiang, China). Cyclophosphamide was purchased from Shanghai Ruiyong Biotechnology Co., Ltd. (Shanghai, China). Levamisole hydrochloride was purchased from Shanghai Aladdin Biochemical Technology Co., Ltd. (Shanghai, China). Mouse tumor necrosis factor-α (TNF-α), interleukin-2 (IL-2), interleukin-4 (IL-4), and interleukin-10 (IL-10) ELISA kit were obtained from Shanghai Jianglai Biotechnology Co., Ltd., (Shanghai, China). ELISA kits for immunoglobulin (IgA, IgG, IgM, and sIgA) and SOD, MDA, and CAT were bought from Nanjing Jiancheng Biological Co., Ltd., (Nanjing, China).

### 2.2. Screening Methodology

#### 2.2.1. In Vitro Antioxidant Activity

(1) 2,2-diphenyl-1-picrylhydrazyl (DPPH) Radical Scavenging Assay

First, 0.5 mL of the enzymatic hydrolysate was mixed with 0.5 mL of pre-prepared 0.5 mmol/L DPPH-ethanol solution, and the reaction was carried out in the dark for 30 min. The absorbance (A_1_) was measured at 517 nm. For the control group, absolute ethanol was used to replace the DPPH solution, and the corresponding absorbance (A_2_) was determined. For the blank group, absolute ethanol was used instead of the sample solution, and the corresponding absorbance (A_0_) was measured. The scavenging rate was calculated as Scavenging Rate (%) = [1 − (A_1_ − A_2_)/A_0_] × 100%.

(2) Superoxide Anion Radical (O_2_^−^) Scavenging Assay

The prepared Tris-HCl buffer was preheated in a 25 °C water bath for 20 min. Then, 4.5 mL of the preheated Tris-HCl buffer was taken, followed by the addition of 1.0 mL of enzymatic hydrolysate and 0.4 mL of 25 mmol/L pyrogallol solution. The mixture was reacted at 25 °C for 4 min, after which 1.0 mL of 8% HCl was added to terminate the reaction. The absorbance (A_1_) was measured at 320 nm. For the control group, deionized water was used to replace the pyrogallol solution, and the corresponding absorbance (A_2_) was determined. For the blank group, deionized water was used instead of the sample solution, and the corresponding absorbance (A_0_) was measured. The scavenging rate was calculated as Scavenging Rate (%) = [1 − (A_1_ − A_2_)/A_0_] × 100%.

(3) Hydroxyl radical (·OH) scavenging capacity

First, 0.5 mL of enzymatic hydrolysate, 0.5 mL of 6 mmol/L salicylic acid ethanol solution, and 0.5 mL of 6 mmol/L ferrous sulfate solution were mixed thoroughly, followed by the addition of 0.5 mL of 3% hydrogen peroxide solution. The mixture was reacted at 37 °C for 30 min. The absorbance of the sample (A_1_) was measured at 510 nm. For the control group, an equal volume of deionized water was used to replace the hydrogen peroxide solution, and the corresponding absorbance (A_2_) was determined. For the blank group, an equal volume of deionized water was used instead of the enzymatic hydrolysate, with the absorbance recorded (A_0_). The scavenging rate was calculated as Scavenging Rate (%) = [1 − (A_1_ − A_2_)/A_0_] × 100%.

#### 2.2.2. Determination of Degree of Hydrolysis (DH)

The DH was measured using the o-phthaldialdehyde (OPA) method. Briefly, 0.2 mL of enzymatic hydrolysate was mixed with 2.0 mL OPA reagent, incubated at 37 °C for 2 min, and absorbance was read at 340 nm. A standard curve was prepared with L-leucine, and DH was calculated as DH (%) = [(h − h_0_)/(h_1_ − h_0_)] × 100, where h is the absorbance of the sample, h_0_ is that of the unhydrolyzed protein, and h1 is the maximum absorbance from fully hydrolyzed protein.

### 2.3. Obtainment of Antioxidant Polysaccharides and Polypeptides

#### 2.3.1. Flowchart

**Figure 1:**
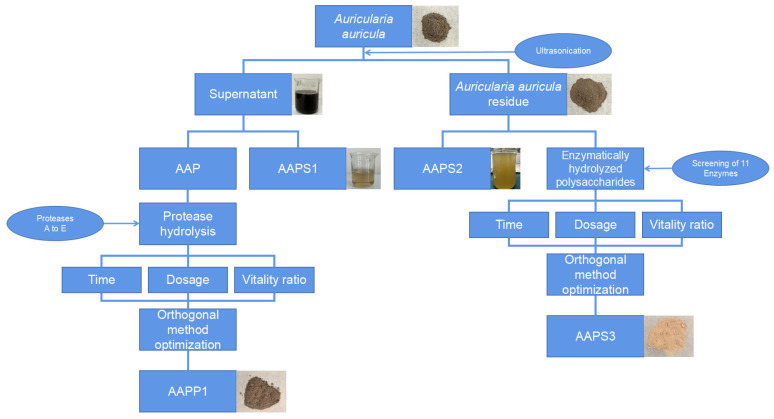
Extraction process of Auricularia auricula protein, polypeptide, and polysaccharide.

The preparation procedures of *Auricularia auricula* polypeptides and polysaccharides are shown in Figure 1. First, the fruiting bodies of *Auricularia auricula* were subjected to protein extraction using an ultrasonic method. The resulting protein solution was then separated into *Auricularia auricula* protein and water-soluble polysaccharides via ammonium sulfate precipitation. The *Auricularia auricula* protein was further subjected to enzymatic hydrolysis to obtain polypeptides, with concurrent optimization of the enzymatic hydrolysis process.

The residual *Auricularia auricula* residues after ultrasonic protein extraction were subjected to water extraction to obtain water-extracted polysaccharides. Meanwhile, these residues were also subjected to enzymatic hydrolysis to obtain *Auricularia auricula* polysaccharides, with optimization of the enzymatic hydrolysis process performed simultaneously.

#### 2.3.2. Extraction of Auricularia Auricula Protein (AAP)

The AAP was extracted under the conditions of a solid–liquid ratio of 1:50 g/mL, ultrasonic power of 350 W, pH 10, and ultrasonic time of 1.5 h. After centrifugation, the supernatant was collected. The residue was subjected to a second extraction under the same conditions. The two extraction supernatants were combined, and proteins were precipitated with (NH_4_)_2_SO_4_ at a final concentration of 45%, followed by lyophilization. The *Auricularia auricula* residue after two extractions was lyophilized for subsequent use.

#### 2.3.3. Obtainment of Antioxidant Polypeptides and Polysaccharides

(1) Antioxidant Polypeptides

a. Enzymatic Hydrolysis of Polypeptides

The AAP was mixed with distilled water at a fixed ratio, and proteases A to E were separately added. The enzyme dosage was set at 7000 U/g, with a hydrolysis time of 120 min, stirring at 70 r/min. Other conditions were optimized for each of the five enzymes’ respective optimal activities. After enzymatic hydrolysis, the mixture was subjected to a boiling water bath for 10 min to inactivate the enzymes. Once the hydrolysate cooled to room temperature, it was centrifuged at 10,000 r/min for 10 min, and the supernatant was collected. The in vitro antioxidant activities (DPPH, O_2_^−^ scavenging capacity, and ·OH scavenging capacity) and protein DH were measured. Single enzymes with better performance were pairwise combined, and the optimal compound enzyme combination was obtained under the same screening conditions. The types of proteases are listed in Table 1.

b. Optimization of Enzymatic Hydrolysis Conditions

Using the DPPH scavenging rate of the enzymatic hydrolysis product as an index, the effects of hydrolysis time (60, 90, 120, 150, 180 min), enzyme dosage (5000, 6000, 7000, 8000, 9000 U/g), and the activity ratio of protease B to protease E (1:2, 1:1.5, 1:1, 1.5:1, 2:1) were investigated. The resulting polypeptide was designated as AAPP1. Based on this, an orthogonal test was designed to optimize the enzymatic hydrolysis conditions by selecting three factors: the activity ratio of protease B to protease E (A), enzyme dosage (B), and hydrolysis time (C). The factor levels are shown in Table 2.

(2) Antioxidant Polysaccharides

a. Polysaccharides in Supernatant from Ammonium Sulfate Precipitation of Protein (AAPS1)

The supernatant generated during the ammonium sulfate precipitation of AAP in Section 2.2.1 was subjected to rotary evaporation for a concentration of 1/5 of its original volume. Precipitated ammonium sulfate solids were filtered using a Büchner funnel. The solution was then placed in a dialysis bag and dialyzed against running water for 36 h. The dialyzed solution was concentrated, followed by alcohol precipitation and lyophilization.

b. Preparation of Water-Extracted Polysaccharides from *Auricularia auricula* Residue (AAPS2)

The *Auricularia auricula* residue obtained in Section 2.2.1 was mixed at a solid–liquid ratio of 1:50, extracted by immersion at 100 °C for 120 min, and centrifuged at 10,000 r/min for 10 min. The supernatant was concentrated to 1/3 of its volume, followed by alcohol precipitation and lyophilization.

c. Enzymatically Hydrolyzed Polysaccharides (AAPS3)

The *Auricularia auricula* residue obtained in Section 2.2.1 was mixed with distilled water at a solid–liquid ratio of 1:50 (g/mL). Enzymes 1 to 11 were separately added, with an enzyme dosage set at 10,000 U/g, hydrolysis time of 120 min, and stirring at 70 r/min. Other conditions were optimized for each of the 11 enzymes’ respective optimal activities. After enzymatic hydrolysis, the mixture was boiled in a water bath for 10 min to inactivate the enzymes. Once the hydrolysate cooled to room temperature, it was centrifuged at 10,000 r/min for 20 min, and the supernatant was collected. The in vitro antioxidant activity and polysaccharide content were measured. Single enzymes with better performance were pairwise combined, and the optimal compound enzyme combination was obtained under the same screening conditions. The types of the 11 enzymes are listed in Table 3.

d. Optimization of Enzymatic Hydrolysis Conditions

Using the in vitro antioxidant activity and polysaccharide content of the enzymatic hydrolysis products as indicators, the effects of hydrolysis time (30, 60, 90, 120, 150 min), enzyme dosage (1000, 5000, 10,000, 15,000, 20,000 U/g), and the activity ratio of Enzyme 2:Enzyme 5 (1:2, 1:1.5, 1:1, 1.5:1, 2:1) were investigated. The enzymatically hydrolyzed polysaccharides obtained were denoted as AAPS3. Based on this, three factors—hydrolysis time (A), enzyme dosage (B), and activity ratio (C)—were selected to design an orthogonal test for optimizing the enzymatic hydrolysis conditions. The factor levels are shown in Table 4.

### 2.4. Animal Model and Study Design

Forty male Kunming mice (6–8 weeks old, specific pathogen-free), weighing 20 ± 2 g, were randomly divided into five groups: Group Control (Con), Group Model (Mod), Group Levamisole hydrochloride (PC), Group AAPP1, and Group AAPS3, with 8 mice per group. All mice were acclimatized for 7 days before modeling. Body weight (BW) was measured at fixed time points. All mice were administered drugs by gavage at a volume of 0.1 mL/10 g once daily at fixed time points for 2 consecutive weeks. The grouping and administration details are shown in Table 5.

### 2.5. Determination of the Immune Organ Indices

The thymus and spleen were excised and weighed. The immune organ indices were calculated using the following formula: Immune organ index (mg/g) = immune organ weight (mg)/body weight (g).

### 2.6. Histopathological Analysis of Spleen

Referring to the method of [20] with slight modifications, mouse spleen tissues were harvested and immediately immersed in 4% paraformaldehyde solution for fixation. Following fixation, the tissues were dehydrated through a graded ethanol series, embedded in paraffin using standard protocols, and sectioned at a thickness of 5 μm. Hematoxylin-eosin (HE) staining was performed, and the pathological changes in spleen tissues were observed under a light microscope.

### 2.7. Gut Microbiota Analysis of Feces

Mice were placed in clean and sterile collection cages with sterile collection plates pre-placed at the bottom. After the mice defecated naturally, fresh feces were promptly collected using sterile forceps. The collected fecal samples were immediately transferred into sterile 1.5 mL centrifuge tubes and rapidly subjected to quick-freezing treatment in dry ice to maximize the preservation of the original state of the intestinal flora, thereby avoiding changes in the composition and abundance of the flora. Subsequently, the centrifuge tubes containing fecal samples were transferred to a −80 °C ultra-low temperature refrigerator for long-term storage until intestinal flora analysis was performed. The sequencing of gut microbiota was commissioned to Nanjing Paisenuo Gene Technology Co., Ltd., Nanjing, China.

#### 2.7.1. DNA Extraction

Total genomic DNA samples were extracted using the OMEGA Soil DNA Kit (M5635-02) (Omega Bio-Tek, Norcross, GA, USA) for feces following the manufacturer’s instructions and stored at −20 °C prior to further analysis. The quantity and quality of extracted DNAs were measured using a NanoDrop NC2000 spectrophotometer (Thermo Fisher Scientific, Waltham, MA, USA) and agarose gel electrophoresis, respectively.

#### 2.7.2. 16S rRNA Gene Amplicon Sequencing

PCR amplification of the bacterial 16S rRNA genes V3–V4 region was performed using the forward primer 338F (5′-ACTCCTACGGGAGGCAGCA-3′) and the reverse primer 806R (5′-GGACTACHVGGGTWTCTAAT-3′). Sample-specific 7 bp barcodes were incorporated into the primers for multiplex sequencing. The PCR components contained 5 μL of buffer (5×), 0.25 μL of fast pfu DNA polymerase (5 U/μL), 2 μL (2.5 mM) of dNTPs, 1 μL (10 μM) of each forward and reverse primer, 1 μL of DNA template, and 14.75 μL of ddH2O. Thermal cycling consisted of initial denaturation at 98 °C for 5 min, followed by 25 cycles consisting of denaturation at 98 °C for 30 s, annealing at 53 °C for 30 s, and extension at 72 °C for 45 s, with a final extension of 5 min at 72 °C. PCR amplicons were purified with Vazyme VAHTSTM DNA Clean Beads (Vazyme, Nanjing, China) and quantified using the Quant-iT PicoGreen dsDNA Assay Kit (Invitrogen, Carlsbad, CA, USA). After the individual quantification step, amplicons were pooled in equal amounts, and pair-end 2 × 300 bp sequencing was performed using the Illlumina MiSeq platform with MiSeq Reagent Kit v3 at Shanghai Personal Biotechnology Co., Ltd. (Shanghai, China).

#### 2.7.3. Sequence Analysis

Microbiome bioinformatics were performed with QIIME2 2022.11 with slight modification according to the official tutorials. Briefly, raw sequence data were demultiplexed using the demux plugin following by primers cutting with the cutadapt plugin. Sequences were then quality filtered, denoised, merged, and chimera removed using the DADA2 plugin. Non-singleton amplicon sequence variants (ASVs) were aligned with mafft and used to construct a phylogeny with fasttree2.

### 2.8. Determination of the Immunoglobulin Contents

To collect serum, whole blood was centrifuged at 3500 rpm for 10 min. For the small intestine sample, approximately 60 mg of intestinal tissue was accurately weighed, homogenized with sterile saline, and then centrifuged at 3500 rpm for 15 min; the resulting supernatant was collected for subsequent assays. The contents of immunoglobulins in both serum and small intestine samples were determined using commercial assay kits (Nanjing Jiancheng Biological Co., Ltd.) strictly following the manufacturer’s standardized operating protocols.

### 2.9. Determination of the Level of Oxidative Stress in the Liver

Liver oxidative stress in the different groups was assessed by measuring malondialdehyde (MDA), superoxide dismutase (SOD), and catalase (CAT) levels using commercial kits obtained from Jiancheng Creatures Co. Ltd. (Nanjing, China).

### 2.10. Statistical Analysis

The results are expressed as ‘Mean ± SD’. One Way ANOVA was performed using IBM SPSS Statistics 21 software for comparison, and Origin 2021 software and the Paisenno gene cloud platform were used for plotting. A heatmap was plotted by https://www.bioinformatics.com.cn (last accessed on 10 December 2024), an online platform for data analysis and visualization.

## 3. Result and Discussion

### 3.1. Screening of Optimal Conditions to Prepare AAPP1

a. Screening of Proteases

**Figure 2:**
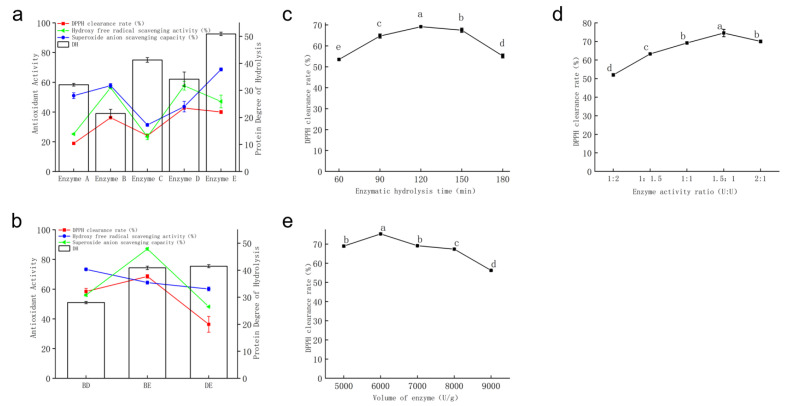
Preparation of polypeptides by enzymatic hydrolysis. (**a**) Screening of single proteases; (**b**) screening of protease compound combinations; (**c**) optimization of hydrolysis time; (**d**) optimization of compound enzyme ratio; (**e**) enzyme dosage. Values are expressed as mean ± standard deviation (*n* = 3 independent experiments). Different superscript letters indicate significant differences (*p* < 0.05) determined by one-way ANOVA followed by Duncan’s test.

The effects of five proteases on DH and in vitro antioxidant activity of AAP are shown in Figure 2a. Enzyme D (42.68%) and enzyme E (40.00%) exhibited higher DPPH radical scavenging rates, while enzyme A (18.88%) and enzyme C (24.11%) showed relatively close DPPH scavenging rates. Enzyme B (57.73%) and enzyme E (68.66%) demonstrated stronger ·OH radical scavenging rate. In contrast, the ·OH scavenging rate (23.37%) and O_2_^−^ scavenging rate (31.33%) of enzyme C’s hydrolysate were lower than those of the other four proteases. The antioxidant activity of enzyme B’s hydrolysate outperformed that of enzyme A, consistent with the findings of He [21]. Kim et al. [22] reported that three antioxidant peptides isolated from Halocynthia roretzi protein hydrolyzed by enzyme D showed higher Fe^2+^ chelating activity than glutathione (GSH). Enzyme E can hydrolyze proteins to generate proline residues, a hydrophobic amino acid [23]. Research has indicated that peptides with hydrophobic or aromatic amino acids at their terminals exhibit higher antioxidant activity [24].

A comprehensive evaluation of the in vitro antioxidant activities of the five proteases showed the following efficacy hierarchy: enzyme D > enzyme E > enzyme B > enzyme A > enzyme C. Enzymes B, D, and E exhibited stronger in vitro antioxidant activities. As combinatorial enzymatic hydrolysis for protein hydrolysis can achieve a synergistic effect [25], enzymes B, D, and E were selected for pairwise combination to screen for the optimal combined enzyme mixture. Compared with enzymes D and E, the DH of AAP by enzyme B was the lowest. Therefore, enzyme B was selected as the first-step enzyme for BD and BE combinations. When comparing enzymes D and E, enzyme D had a lower degree of protein hydrolysis, so enzyme D was chosen as the first-step enzyme for the DE combination. As shown in Figure 2a, there was minimal correlation between the antioxidant activity of the enzymatic hydrolysates of different proteases and their degrees of hydrolysis. The possible reason may be that enzymes have specificity, dictating differences in the composition and structure of the hydrolysates during the enzymatic hydrolysis process. These differences consequently result in variations in their electron-donating or hydrogen-donating capabilities, increasing differences in their antioxidant activities.

b. Screening of Protease Compound Combinations

The results of combined enzymatic treatments BD, BE, and DE are shown in Figure 2b. Enzyme BE (68.58%) exhibited a significantly higher DPPH scavenging rate, which was 89% higher than that of enzyme B alone (36.22%) and 67% higher than that of enzyme E alone (40.00%). The order of in vitro antioxidant activities of the three protease combinations was enzyme BE > enzyme BD > enzyme DE, confirming a synergistic effect in protease compounding. As a result, the protease BE combination was selected for subsequent experiments, and the resulting polypeptide was named AAPP1.

c. Optimization of Enzymatic Hydrolysis Conditions

(I) Enzymatic Hydrolysis Time

The influence of hydrolysis time on the DPPH scavenging rate is shown in Figure 2c. Within the 60–120 min interval, the high abundance of AAP in the solution provided sufficient enzymatic cleavage sites. As the hydrolysis time extended, the yield of high-activity antioxidant peptides decreased, and the growth rate of the DPPH scavenging rate of AAPP1 slowed down. The maximum DPPH scavenging rate (69.17%) was achieved at 120 min, while the rates at 150 min (67.47%) and 180 min (55.18%) decreased, possibly due to excessive hydrolysis, where partial polypeptides were broken down into smaller fragments or amino acids, destroying the active structure of AAPP1 and reducing its antioxidant activity. Based on these findings, hydrolysis times of 90 min, 120 min, and 150 min were selected for orthogonal experiments.

(II) Compound Enzyme Ratio

The effect of different compound enzyme ratios on the DPPH scavenging rate is shown in Figure 2d. With the increase in the proportion of protease B, the antioxidant activity of AAPP1 was first enhanced and then diminished. The maximum DPPH scavenging rate (74.53%) was achieved when the enzyme activity ratio of B:E was 1.5:1. He et al. [21] found that the hydrolysate of enzyme B had stronger free radical scavenging ability than those of other proteases. Comprehensively, enzyme activity ratios of 1:1, 1.5:1, and 2:1 were selected for orthogonal testing.

(III) Enzyme Dosage

The effect of enzyme dosage on the DPPH scavenging rate is shown in Figure 2e. The maximum DPPH scavenging rate of AAPP1 (75.32%) was observed at an enzyme dosage of 6000 U/g. With the increase in protease dosage, the binding probability between proteases and substrates increased per unit time. However, when the enzyme was in excess, polypeptides might form complexes with the enzyme [26], inhibiting polypeptide activity and reducing the antioxidant activity of AAPP1. Comprehensively, enzyme dosages of 5000 U/g, 6000 U/g, and 7000 U/g were selected for orthogonal testing.

(IV) Results of Orthogonal Test

Based on the single-factor experiments, orthogonal tests were conducted according to Table 2. The orthogonal test results are shown in Table 6, and the variance analysis results are presented in Table 7.

As shown in Table 5 and Table 6, the influence order of the three factors on the DPPH scavenging rate of AAPP1 was A (activity ratio, U:U) > B (enzyme dosage, U/g) > C (enzymatic hydrolysis time, min). The optimal process combination for AAP enzymatic hydrolysis was A_2_B_2_C_2_, namely an enzyme activity ratio of 1.5:1, an enzyme dosage of 6000 U/g, and an enzymatic hydrolysis time of 120 min.

### 3.2. Screening of Optimal Condition to Prepare AAPS3

a. Single Enzyme Screening

**Figure 3:**
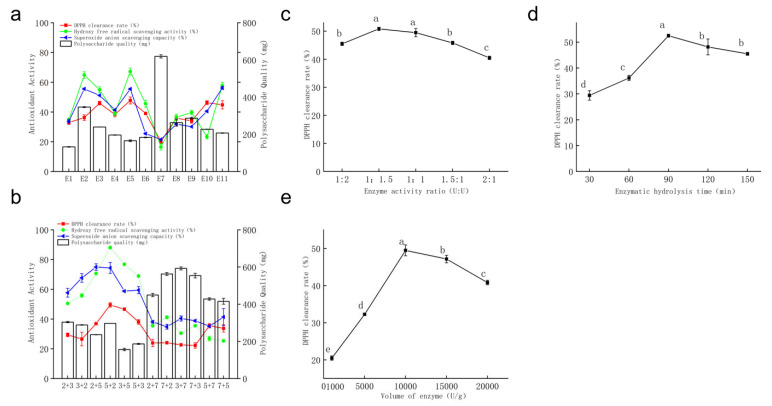
Preparation of polysaccharides by enzymatic hydrolysis. (**a**) Single enzyme screening; (**b**) combined enzyme screening; (**c**) optimization of enzymatic hydrolysis time; (**d**) optimization of combined enzyme ratio; (**e**) enzyme dosage. Values are presented as mean ± standard deviation (*n* = 3 independent experiments). Different superscript letters indicate significant differences determined by one-way ANOVA followed by Duncan’s test (*p* < 0.05).

The in vitro antioxidant activity and polysaccharide content of the enzymatic hydrolysates of *Auricularia auricula* residues by 11 enzymes are shown in Figure 3a. Enzymes 2, 3, and 5 exhibited higher DPPH scavenging rate and ·OH scavenging capacity compared with the other eight enzymes. Among them, the polysaccharide content obtained from enzyme 2-hydrolyzed *Auricularia auricula* residues was higher than that from enzymes 3 and 5. Enzyme 7 showed poor in vitro antioxidant activity among the 11 enzymes, and all three evaluation indices were at the lowest levels (20.12%, 16.53%, 21.49%), but its polysaccharide content was approximately 56% higher than that of enzyme 2, which indicates better polysaccharide dissolution. This may be due to excessive enzymatic hydrolysis causing over-degradation of polysaccharide chains and an increase in low-molecular-weight fragments. Previous studies have reported that low-molecular-weight polysaccharides have more reducing hydroxyl terminals and stronger antioxidant activity [27]. The low antioxidant activity of enzyme 7’s hydrolysate may be attributed to the cleavage of key branched chains by excessive enzymatic hydrolysis, leading to the loss of important active sites in polysaccharides.

Enzymes 1, 6, 8, and 9 showed relatively low in vitro antioxidant activity and polysaccharide content in their hydrolysates. Overall, the in vitro antioxidant activity of the 11 enzymes followed the order enzyme 2 > enzyme 3 > enzyme 5 > enzyme 11 > enzyme 4 > enzyme 6 > enzyme 9 > enzyme 8 > enzyme 1 > enzyme 10 > enzyme 7. Enzymes exhibit high specificity. When multiple enzymes catalyze the same substrate, they can act on a broader range of glycosidic bond types, resulting in relatively smaller molecular weights, which are more easily absorbed by the human body to exert physiological activities. Therefore, enzymes 2, 3, and 5 were selected for pairwise combination. Compared with other enzymes, enzyme 7 could improve polysaccharide yield, so it was also combined with the above enzymes for comparison.

b. Combined Enzyme Screening

Pairwise combinations of enzymes were evaluated, and the results are shown in Figure 3b. Combinations involving enzyme 7 (2 + 7, 7 + 2, 3 + 7, 7 + 3, 5 + 7, 7 + 5) significantly increased polysaccharide content, but all three in vitro antioxidant activities were relatively low. Additionally, Figure 3b shows that different enzyme addition orders had little effect on polysaccharide activity, but different enzyme combinations influenced polysaccharide dissolution rates. The combined enzyme 5 + 2 demonstrated the highest in vitro antioxidant activity among the 12 combinations, with a DPPH scavenging rate of 49.47%, ·OH scavenging capacity of 88.05%, and O_2_^−^ scavenging capacity of 74.36%. Thus, the enzyme combination of 5 + 2 was selected for subsequent experiments, and the resulting polysaccharide was designated as AAPS3.

c. Optimization of enzymatic hydrolysis conditions

(I) Enzymatic Hydrolysis Time

The effect of enzymatic hydrolysis time on the DPPH scavenging rate of AAPS3 is shown in Figure 3c. From 30 min to 90 min, the DPPH scavenging rate of AAPS3 gradually increased with prolonged hydrolysis time. At 30 min, the hydrolysis time was insufficient, and the hydrolysate either failed to reach the optimal hydrolysis temperature or ceased shortly after reaching it before stopping, resulting in incomplete hydrolysis of *Auricularia auricula* residues, incomplete fragmentation of polysaccharide molecules, and relatively low DPPH scavenging rate. The maximum activity of AAPS3 (52.49%) was achieved at 90 min. Prolonging the hydrolysis time further led to a decrease in the DPPH scavenging rate, possibly due to the destruction of polysaccharide active structures by excessive hydrolysis or the inhibition of enzyme activity by hydrolysates from *Auricularia auricula* residues during prolonged hydrolysis [28,29]. Therefore, 60, 90, and 120 min were selected for orthogonal experiments.

(II) Proportion of Combined Enzymes

The effect of different combined enzyme proportions on the DPPH scavenging rate of AAPS3 is shown in Figure 3d. As the activity proportion of enzyme 2 increased, the antioxidant activity of AAPS3 first increased and then decreased. The maximum DPPH scavenging rate (50.83%) was achieved at an enzyme 2–enzyme 5 ratio of 1:1.5. Enzyme 2 can cleave α-1,4 glycosidic bonds, and increasing the concentration of enzyme 2 led to an increase in glucose residue terminals in the hydrolysate, thereby enhancing antioxidant activity. Based on these results, enzyme activity ratios of 1:2, 1:1.5, and 1:1 were selected for orthogonal experiments.

(III) Enzyme Dosage

The effect of enzyme dosage on the DPPH scavenging rate of AAPS3 is shown in Figure 3e. With increasing enzyme dosage, the DPPH scavenging rate of AAPS3 first increased and then decreased, peaking at 49.47% at an enzyme dosage of 10,000 U/g. Increasing enzyme dosage can enhance the contact area between substrate and enzyme, which accelerates the reaction rate. When the enzyme–substrate ratio reached saturation, increasing the enzyme dosage did not lead to more reactions due to substrate limitation. Additionally, according to enzyme kinetics, excessively high enzyme concentrations can cause enzyme–enzyme aggregation, inhibiting enzymatic activity [26] and reducing hydrolysis efficiency. Consequently, enzyme dosages of 5000; 10,000; and 15,000 U/g were selected for orthogonal experiments.

(IV) Results of Orthogonal Test

Based on the single-factor experiments, orthogonal tests were conducted according to Table 4. The orthogonal test results are shown in Table 8, and the variance analysis is presented in Table 9.

As shown in Table 7 and Table 8, the order of influence of the three factors on the DPPH scavenging rate of AAPS3 was A (enzymatic hydrolysis time, min) > C (activity ratio, U:U) > B (enzyme dosage, U/g). The optimal technological combination of enzymatic hydrolysis conditions was A_2_B_2_C_1_, namely an enzyme activity ratio of 1:2, an enzyme dosage of 10,000 U/g, and an enzymatic hydrolysis time of 90 min.

### 3.3. Comparison of In Vitro Antioxidant Activities of Each Component

The half maximal inhibitory concentration (IC_50_) values of each component extracted from *Auricularia auricula* are shown in Table 10. The order of antioxidant activity among the components was AAPS3 > AAPP1 > AAPS1 > AAPS2 > AAPP. The IC_50_ value of DPPH scavenging rate for enzymatic hydrolysate polysaccharide from *Auricularia auricula* residues (AAPS3) was 0.53 mg/mL, which was 56.55% lower than that of water-extracted polysaccharides from *Auricularia auricula* residues (AAPS2, IC_50_ = 1.22 mg/mL). The ·OH scavenging capacity and O_2_^−^ scavenging capacity of AAPS3 were 47.98% and 50.54% lower, respectively, than those of AAPS2. For *Auricularia auricula* antioxidant peptides (AAPP1), the IC_50_ value of DPPH scavenging rate was 0.63 mg/mL, representing a 66.49% reduction compared to *Auricularia auricula* protein (AAP, IC_50_ = 1.88 mg/mL), with ·OH scavenging capacity and O_2_^−^ scavenging capacity decreasing by 46.35% and 14.03%, respectively. Additionally, Table 9 shows that the activity of enzymatic hydrolysis polysaccharides (AAPS3) > ultrasonic extraction polysaccharides (AAPS1) > water extraction polysaccharides (AAPS2).

### 3.4. Effects of AAPP1 and AAPS3 on Body Weight and Immune Organ Indexes of Immunosuppressed Mice

a. Body weight and hair changes in immunosuppressed mice

**Figure 4:**
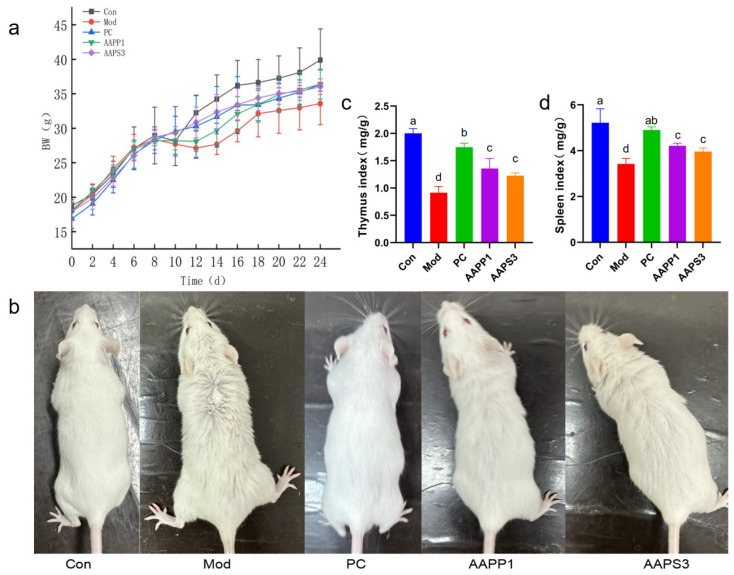
Effects of AAPP1 and AAPS3 supplementation on immunosuppressed mice. (**a**) Changes in mouse body weight; (**b**) hair changes in each group of mice after 7 days of gavage; (**c**) thymus index; (**d**) spleen index. Values are presented as mean ± standard deviation (*n* = 8 independent experiments). Different superscript letters indicate significant differences determined by one-way ANOVA followed by Duncan’s test (*p* < 0.05).

This study recorded changes in mouse body weight over 24 days from the start of adaptive culture and CTX injection to the end of gavage. Initially, the average body weight of mice in each group was similar, but after CTX injection, body weight decreased compared with Group Con, preliminarily confirming the successful establishment of the immunodeficiency model. After intervention with PC, AAPP1, and AAPS3, mouse body weight recovered compared with Group Mod, as shown in Figure 4a.

Differences in mouse hair were observed among groups due to intraperitoneal CTX injection (or 0.9% saline) and gavage (or 0.9% saline). As shown in Figure 4b, all groups except Group Con exhibited hair loss, with the most obvious hair loss in Group Mod. Hair loss was improved in mice gavaged with PC, AAPP1, and AAPS3. The results indicate that PC, AAPP1, and AAPS3 can alleviate adverse symptoms caused by CTX.

b. Effects on Immune Organ Indexes of Immunosuppressed Mice

As shown in Figure 3c,d, compared with Group Con, Group Mod had lower thymus and spleen indexes, indicating successful establishment of the immunosuppressed mouse model by 3-day CTX injection. The thymus and spleen are important lymphoid organs in animals, serving as sites for immune cell development and maturation and immune responses. Therefore, immune organ indexes can objectively reflect the body’s nonspecific immune capacity [30,31]. Generally, changes in immune indexes are accompanied by changes in immune organ indexes, so changes in immune organ indexes can be used to judge the effect of drugs on immune activity [32].

After 14 days of gavage, the thymus and spleen indexes of Group PC, Group AAPP1, and Group AAPS3 were significantly higher than those of Group Mod, with comparable effects between AAPS3 and AAPP1 gavage. These results suggest that supplementation with PC, AAPP1, and AAPS3 can improve CTX-induced damage to the thymus and spleen and alleviate CTX-induced splenic and thymic atrophy, consistent with the findings of KX et al. [33].

### 3.5. Effects of AAPP1 and AAPS3 on Serum Cytokines and Immunoglobulins in Immunosuppressed Mice

a. Changes in Serum Cytokines in Immunosuppressed Mice

**Figure 5:**
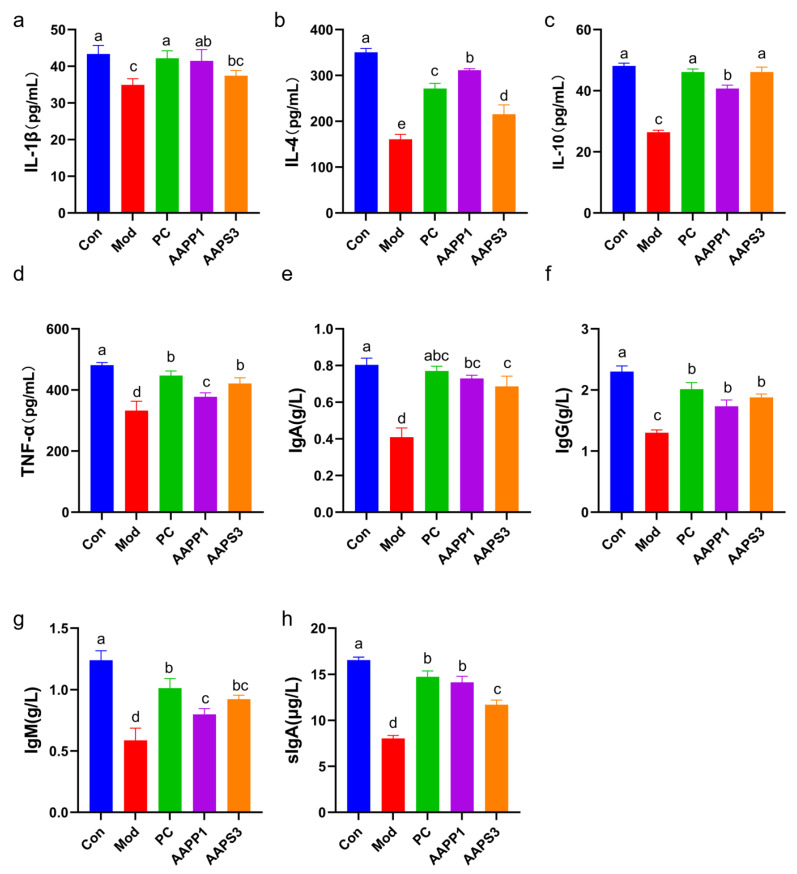
Effects of AAPP1 and AAPS3 supplementation on cytokines and immunoglobulins in immunosuppressed mice. (**a**) IL-1β; (**b**) IL-4; (**c**) IL-10; (**d**) TNF-α; (**e**) IgA; (**f**) IgG; (**g**) IgM; (**h**) sIgA. Values are presented as mean ± standard deviation (*n* = 8 independent experiments). Different superscript letters indicate significant differences determined by one-way ANOVA followed by Duncan’s test (*p* < 0.05).

As shown in Figure 5a–d, the levels of serum IL-1β, IL-4, IL-10, and TNF-α were measured. Compared with Group Con, the serum cytokine levels in Group Mod were significantly decreased (*p* < 0.05), indicating that intraperitoneal CTX injection impaired the immune function of mice. In contrast, the levels of cytokines (IL-4, IL-10, and TNF-α) in Group PC, Group AAPP1, and Group AAPS3 were significantly higher than those in Group Mod (*p* < 0.05). These results suggest that supplementation with PC, AAPP1, and AAPS3 can regulate cytokine levels to improve the immunosuppressive state in mice. Previous studies have confirmed that polypeptides and polysaccharides can enhance immunity by regulating cytokine levels. For example, Yalin Z et al. [34] reported that *Tremella fuciformis* polysaccharides could upregulate the levels of IL-2, IL-12, INF-γ, and IgG in the serum of CTX-induced immunosuppressed mice and significantly promote the mRNA expression of IL-1β and IL-4, exerting a protective effect against CTX-induced immunosuppression. Tingting Yang et al. [35] demonstrated that peptides from Antarctic krill could increase the expression of IL-1, IL-6, IL-10, and TNF-α in the serum of CTX-induced immunosuppressed mice at both the mRNA and protein levels.

b. Changes in Immunoglobulin Content in Serum and Intestinal Tract of Immunosuppressed Mice

The ELISA results for immunoglobulin (IgA, IgG, IgM, sIgA) content are shown in Figure 5e–h. The contents of IgA, IgG, and IgM in the serum of Group Mod were reduced relative to those in Group Con (*p* < 0.05). Compared with Group Mod, the serum contents of IgA, IgG, and IgM in Group PC, Group AAPP1, and Group AAPS3 were significantly increased (*p* < 0.05). Additionally, the intestinal mucosal sIgA content in Group Mod was significantly lower than that in Group Con (*p* < 0.05), while Group PC, Group AAPP1, and Group AAPS3 showed significantly higher intestinal mucosal sIgA content than Group Mod (*p* < 0.05). The effects of Group AAPP1 and Group AAPS3 on IgA, IgG, and sIgA were comparable to those of Group PC. These results indicate that Group PC, Group AAPP1, and Group AAPS3 can restore immune function of CTX-induced immunocompromised mice by regulating humoral immunity. Zhao et al. [36] reported that fructooligosaccharides could enhance the immunity of CTX-induced immunosuppressed mice by regulating immunoglobulins.

### 3.6. Protective Effects of AAPP1 and AAPS3 on Oxidative Damage in Liver Tissues of Immunosuppressed Mice

**Figure 6:**
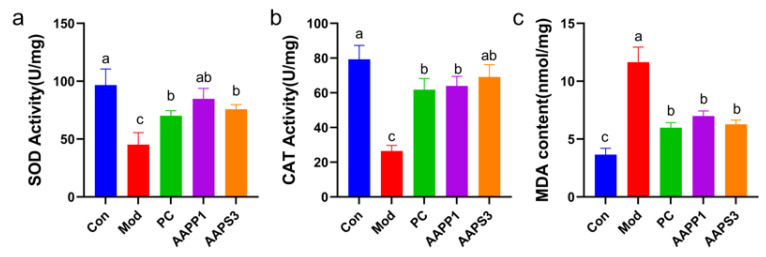
Effects of AAPP1 and AAPS3 supplementation on antioxidant enzyme activities in liver cells of immunosuppressed mice. (**a**) SOD; (**b**) CAT; (**c**) MDA. Values are presented as mean ± standard deviation (*n* = 8 independent experiments). Different superscript letters indicate significant differences determined by one-way ANOVA followed by Duncan’s test (*p* < 0.05).

As shown in Figure 6a–c, compared with Group Con, the activities of SOD and CAT in Group Mod were significantly decreased (*p* < 0.05). Gavage with PC, AAPP1, and AAPS3 significantly increased the activities of SOD and CAT in mouse liver tissues, restored hepatic antioxidant capacity, and reduced the content of MDA in the liver. SOD, a key antioxidant enzyme in living organisms, specifically catalyzes the disproportionation of superoxide anion radicals into hydrogen peroxide and oxygen, thereby scavenging intracellular superoxide anions and alleviating oxidative stress-induced damage to hepatocytes [37,38]. CAT decomposes hydrogen peroxide generated by SOD into water and oxygen, forming a hydrogen peroxide scavenging system with glutathione peroxidase to prevent intracellular accumulation of toxic hydrogen peroxide and further protect hepatocytes from oxidative damage [39,40]. MDA, as the end product of lipid peroxidation, indicates aggravated oxidative damage to hepatocytes when its level increases [41].

### 3.7. Histopathology Examination

**Figure 7:**
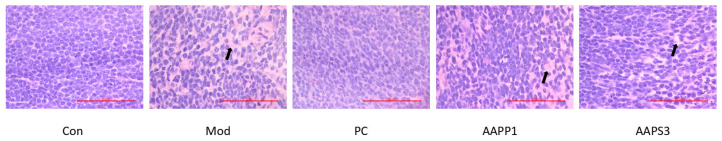
Effects of AAPP1 and AAPS3 supplementation on histological changes in the spleen of immunosuppressed mice. The arrow indicates an area of loosely arranged cells.

As shown in Figure 7, in the Con group, lymphocytes in the white pulp area of the spleen were densely arranged, with uniform morphology and a complete immune structure, reflecting a normal immune function state. In the Mod group, the boundary between the red and white pulp in the mouse spleen was relatively blurred, and the cells were arranged rather loosely, as indicated by the arrow in the figure. In the PC group, AAPP1 group, and AAPS3 group, an increase in cell density and an improvement in structural regularity were observed to varying degrees, and the cell arrangement was relatively regular.

### 3.8. Effects of AAPP1 and AAPS3 on Intestinal Microbiota of Immunosuppressed Mice

a. Analysis of Sample Complexity and Multi-Sample Comparison of Intestinal Microbiota in Mice

(I) α-diversity Analysis

**Figure 8:**
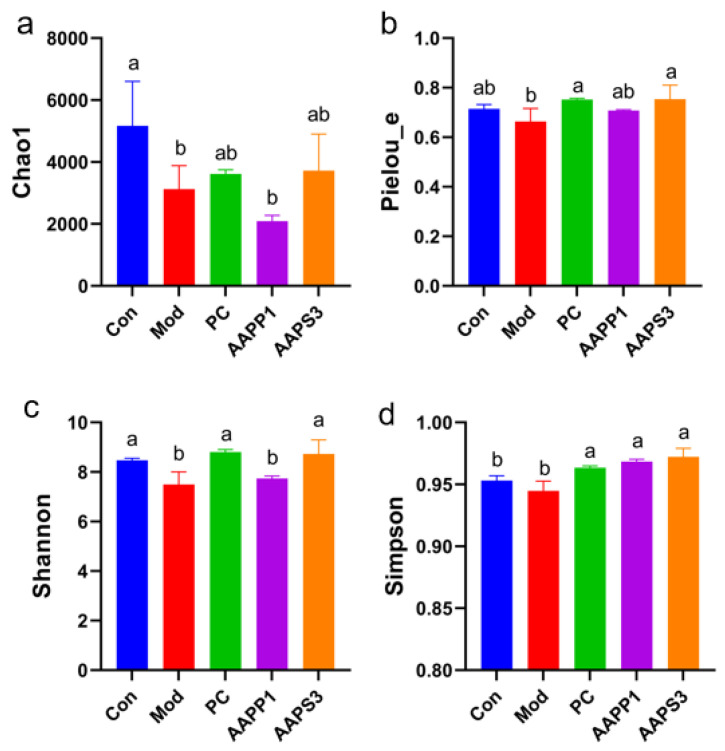
Effects of AAPP1 and AAPS3 supplementation on α-diversity of intestinal microbiota in immunosuppressed mice. (**a**) Chao1; (**b**) Pielou_e; (**c**) Shannon; (**d**) Simpson. Values are presented as mean ± standard deviation (*n* = 3 independent experiments). Different superscript letters indicate significant differences determined by one-way ANOVA followed by Duncan’s test (*p* < 0.05).

Chao1, Simpson, Pielou_e, and Shannon indices were used to characterize the α-diversity of microbial communities, reflecting the diversity of intestinal flora in mice. Chao1 estimates the number of species in community samples, the Simpson index indicates the dominance of species distribution within the community, and the Pielou_e and Shannon indices reflect sample diversity. As shown in Figure 8a–d for intergroup α-diversity analysis, the Chao1 index in Group Mod was significantly lower than that in Group Con (*p* < 0.05), indicating that CTX injection altered the species richness of intestinal microbiota in mice. No significant changes were observed in Group PC and Group AAPS3 compared with Group Con and Group Mod (*p* > 0.05), suggesting that gavage with PC and AAPS3 partially restored the species richness of intestinal flora in mice. The Simpson index in Group PC, Group AAPP1, and Group AAPS3 was significantly higher than that in Group Mod (Figure 8d) (*p* < 0.05), indicating that gavage with AAPP1 and AAPS3 increased species diversity in the mouse intestine. Both Pielou_e and Shannon indices in Group PC and Group AAPS3 showed that continuous gavage with PC and AAPS3 for 14 days reversed CTX-induced decreases in intestinal microbiota diversity and restored it to levels comparable to healthy mice. In summary, continuous gavage with PC, AAPP1, and AAPS3 for 14 days reversed CTX-induced reductions in species richness, abundance, and evenness of intestinal microbiota in immunocompromised mice, restoring them to near-healthy levels, and regulated α-diversity abnormalities of intestinal microbiota in immunocompromised mice. Among them, AAPS3 had a similar effect to PC on α-diversity of intestinal microbiota in immunocompromised mice, which was better than AAPP1.

(II) Veen Diagram

**Figure 9:**
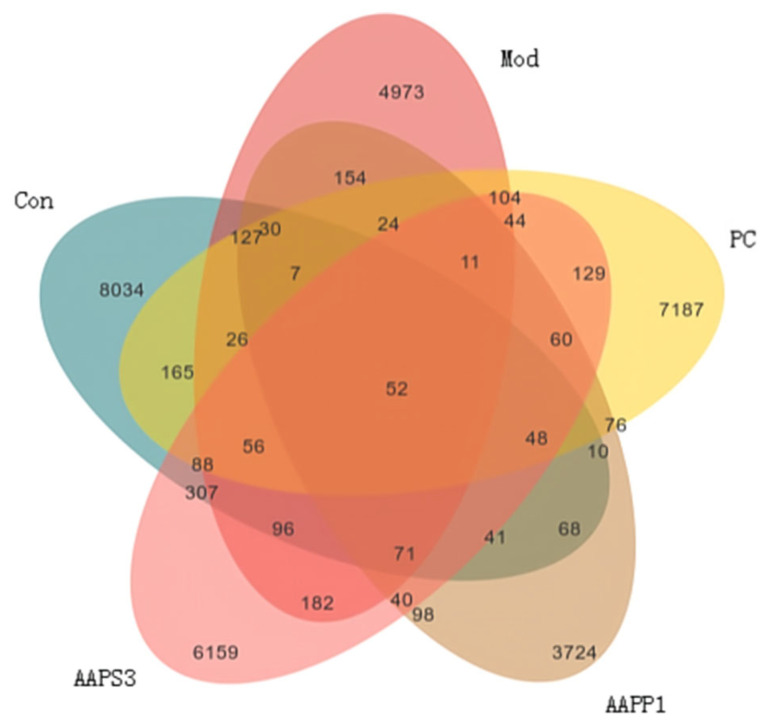
Venn diagram of the effects of AAPP1 and AAPS3 supplementation on the species quantity of intestinal microbiota in immunosuppressed mice.

The Venn diagram (Figure 9) was used to compare the types and quantities of OTUs among different groups, intuitively displaying the similarity and overlap of species composition between groups. As shown in Figure 8, the numbers of unique OTUs in Group Con, Group Mod, Group PC, Group AAPP1, and Group AAPS3 were 8034, 4973, 7187, 3724, and 6159, respectively, with 52 OTUs shared among all five groups. The number of shared OTUs between Group Con and Group Mod was 127, while the shared OTUs between Group Con and Group PC, Group AAPP1, and Group AAPS3 were 165, 68, and 307, respectively. The number of shared OTUs between Group Mod and Group Con was smaller than that between Group PC and Group AAPS3, indicating differences in the composition and structure of intestinal microbiota among groups. Additionally, compared with Group Con, the number of specific OTUs in Group Mod decreased significantly, while Group PC and Group AAPS3 increased the number of specific OTUs to varying degrees, with Group PC showing the largest increase.

b. Analysis of Intestinal Microbiota Structure and Composition in Mice

(I) Phylum Level

**Figure 10:**
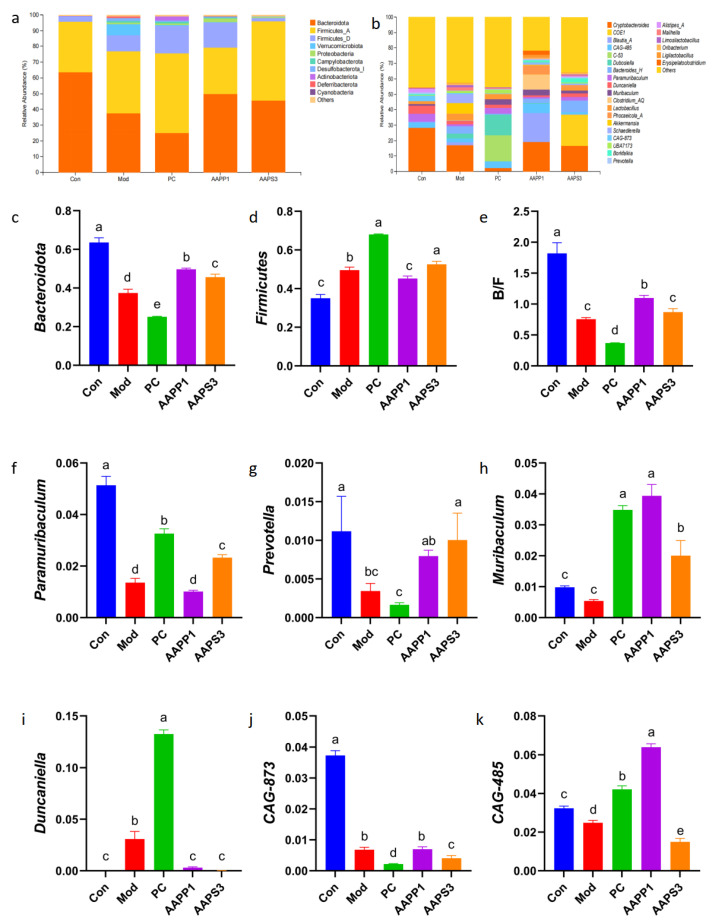
Effects of AAPP1 and AAPS3 supplementation on the species quantity of intestinal microbiota in immunosuppressed mice. (**a**) Phylum level; (**b**) genus level; (**c**) Bacteroidota; (**d**) Firmicutes; (**e**) Bacteroidota/Firmicutes ratio; (**f**) *Paramuribaculum*; (**g**) *Prevotella*; (**h**) *Muribaculum*; (**i**) *Duncaniella*; (**j**) *CAG-873*; (**k**) *CAG-485*. Values are presented as mean ± standard deviation (*n* = 3 independent experiments). Different superscript letters indicate significant differences determined by one-way ANOVA followed by Duncan’s test (*p* < 0.05).

To further investigate the species composition of intestinal microbiota across different groups, we compared differences at the phylum level. Intestinal microbial communities exhibited extremely high species diversity, with species comprising more than 10% of the composition generally considered dominant. At the phylum level (Figure 10a), Bacteroidota and Firmicutes_A were the dominant phyla in Group Con and Group AAPS3, accounting for over 98% of the total microbial community. In Group Mod, Group PC, and Group AAPP1, Bacteroidota, Firmicutes_A, and Firmicutes_D were dominant, with Bacteroidota and Firmicutes accounting for over 87% of the total in Group Mod and over 93% in Group PC and Group AAPP1.

Compared with Group Con, the abundance of Bacteroidota significantly decreased in all groups (*p* < 0.05) (Figure 10c), while Firmicutes (except in Group AAPP1) significantly increased (*p* < 0.05) (Figure 10d), and the Bacteroidota/Firmicutes ratio significantly decreased (*p* < 0.05) (Figure 10e). Gavage with AAPP1 and AAPS3 increased the abundance of Bacteroidota (*p* < 0.05) and elevated the Bacteroidota/Firmicutes ratio, bringing it closer to the level of Group Con. The Bacteroidota/Firmicutes ratio is generally considered to be negatively correlated with in vivo inflammation levels [42]. It is speculated that gavage with AAPP1 and AAPS3 may reduce inflammation in immunocompromised mice by increasing the Bacteroidota/Firmicutes ratio, thereby alleviating CTX-induced inflammation.

(II) Genus Level

As shown in Figure 10, the top 20 abundant genera in the mouse intestine were compared at the genus level. *Cryptobacteroides* was the dominant genus in Group Con and Group Mod; *CAG-873*, *CAG-485*, and *Clostridium_AQ* were dominant in Group PC; *Duncaniella* and *Clostridium_AQ* were dominant in Group AAPP1; and *Cryptobacteroides* and *Clostridium_AQ* were dominant in Group AAPS3.

Compared with Group Con, the abundances of *Paramuribaculum* and *Prevotella* in Group Mod significantly decreased (*p* < 0.05). In contrast, the abundances of *Paramuribaculum* (except in Group AAPS3) and *Muribaculum* in Group PC, Group AAPP1, and Group AAPS3 significantly increased compared with Group Mod (*p* < 0.05). The abundance of *Duncaniella* in Group PC was extremely significantly higher than that in Group Con, Group AAPP1, and Group AAPS3 (*p* < 0.01). The genus *CAG-873* showed extremely high abundance in Group Con (*p* < 0.01).

Studies have shown that *Paramuribaculum* is significantly positively correlated with TNF-α levels in mouse serum and can produce short-chain fatty acids (SCFAs) to alleviate intestinal microbiota dysbiosis [43]. Gavage with PC, AAPP1, and AAPS3 promoted the growth of *Paramuribaculum* in immunocompromised mice, restoring it to a level comparable to that of Group Con. *Prevotella*, a beneficial bacterium associated with high-fiber diets, plays a key role in regulating glucose metabolism and producing SCFAs [44,45]. Fourteen days of gavage with AAPP1 and AAPS3 increased Prevotella in the intestines of immunocompromised mice, approaching Group Con level, with a significant effect observed in Group AAPS3 (*p* < 0.05). Muribaculum, a major member of the Bacteroidota, exhibits immune-regulatory effects and enhances antitumor immunity in immunotherapy [46]. *Duncaniella* is closely associated with increased oxidative stress and reduced anti-inflammatory capacity [47,48]. It was undetected in Group Con but highly abundant in immunocompromised mice. Gavage with AAPP1 and AAPS3 reduced *Duncaniella* abundance, whereas PC gavage led to its enrichment. Notably, *CAG-873* (a member of *Prevotella*) was highly abundant in Group Con, and increases in *CAG-873* and *CAG-485* can elevate intestinal SCFA levels [49].

In summary, compared with Group Con, the ratio of beneficial to harmful bacteria in Group Mod decreased. Gavage with PC, AAPP1, and AAPS3 improved intestinal microbiota disorders in immunocompromised mice by increasing the proportion of beneficial bacteria and reducing harmful bacteria.

(III) Species Differential Analysis of Immunosuppressed Mice

**Figure 11:**
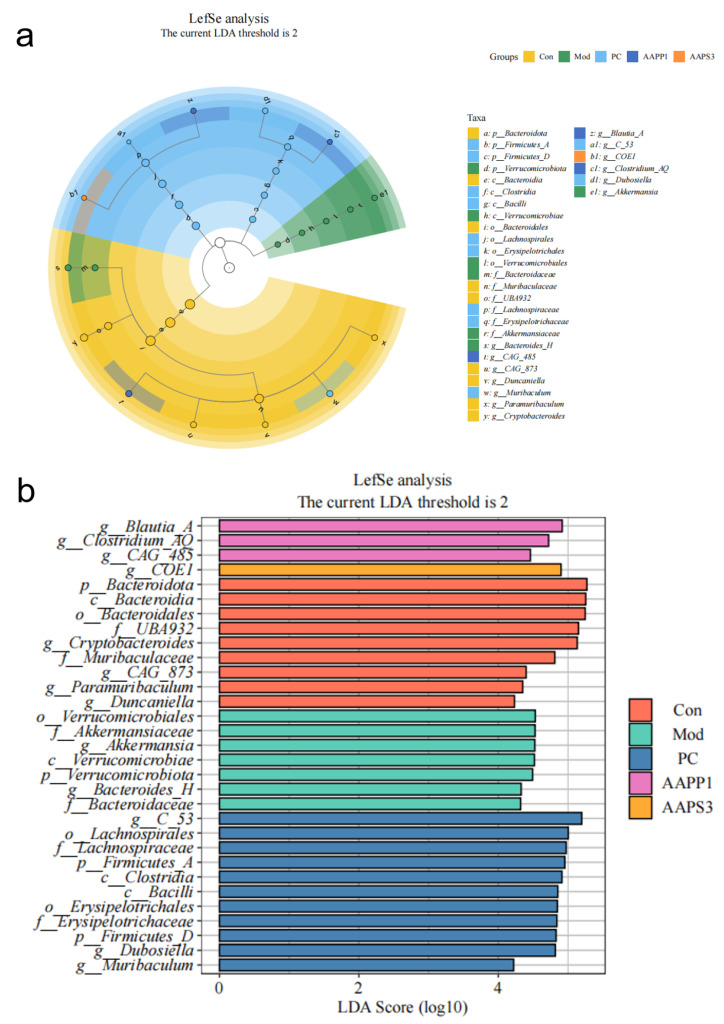
Comparison of linear discriminant analysis effect size (LEfSe) among Group Con, Group Mod, Group PC, Group AAPP1, and Group AAPS3. (**a**) Taxonomic dendrogram and (**b**) LDA histogram (LDA > 2).

Linear discriminant analysis (LDA > 2, abundance filtering threshold 0.01) was used to predict microorganisms with significant effects on the abundance differences of each microbial community (Figure 11). In Group Con, nine microbial groups were significantly enriched, including p-Bacteroidota, c-Bacteeroidia, and o-Bacteroidales. In Group Mod, seven microbial groups were significantly enriched, such as *o-Verrucomicrobiales*, *f-Allermansiaceae*, and *g-Akkermansia.* Group PC had 11 significantly enriched microbial groups, including *g-C-53, o-Lachnospirales*, and *f-Lachnospiraceae*. Group AAPP1 showed significant enrichment of three microbial groups: *g-Blautia-A*, *g-Clostridium-AQ*, and *g-CAG-485*. In the AAPS3 group, *g-COE1* was the significantly enriched microbial group.

### 3.9. Correlation Between the Main Gut Microbiota and the Immune Index

**Figure 12:**
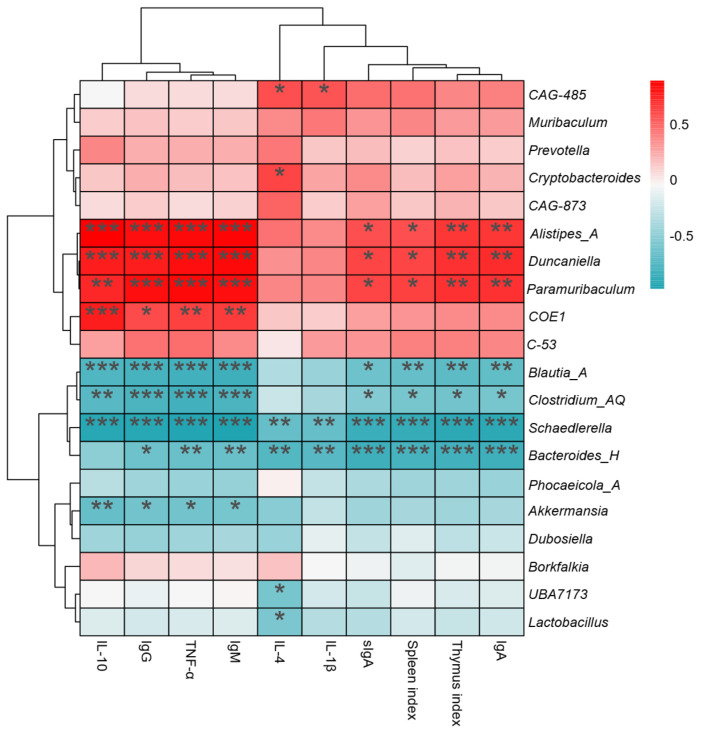
Spearman correlations between genera-level microbiota and immune-related indexes (*** p < 0.001, ** p < 0.01, * p < 0.05).

To explore the relationship between intestinal microbiota and immune function, we performed interactive correlation heatmap analysis(Figure 12). The top 20 abundant communities at the genus level were selected for interactive analysis with immune indexes, serum factors, and immunoglobulins. As shown in Figure 12, the relative abundances of *Bacteroides_H* and *Schaedlerella* were significantly negative correlated with immune indexes, including the thymus index, spleen index, IL-1β, IL-4, TNF-α, IgA, IgG, IgM, and sIgA (*p* < 0.05). *Blautia_A* was significantly negatively correlated with partial immune indexes (*p* < 0.05). In contrast, *Paramuribaculum, Duncaniella, CAG-873*, and *Alistipes_A* showed significant positive correlations with partial immune indexes (*p* < 0.05).

## 4. Conclusions

This study used *Auricularia auricula* as a raw material to achieve step-by-step efficient extraction of polypeptides and polysaccharides from the same batch of raw materials, breaking through the limitations of traditional single-component extraction and significantly improving resource utilization. Polypeptides (AAPP1) and polysaccharides (AAPS3) were extracted step-by-step by a complex enzymatic hydrolysis process to explore their antioxidant and immune-enhancing activities. The results showed that complex enzymatic hydrolysis significantly improved the antioxidant capacity of active components, which was better than traditional extraction methods. In a cyclophosphamide-induced immunosuppressed mouse model, both could significantly improve thymus and spleen indices, upregulate the levels of cytokines such as IL-4 and TNF-α and immunoglobulins such as IgA and IgG, while enhancing the activities of liver SOD and CAT enzymes and reducing the content of MDA, an oxidative damage marker. Intestinal microbiota analysis showed that both could reverse dysbiosis, increase the abundance of beneficial bacteria such as *Paramuribaculum* and *Prevotella,* and reduce the proportion of pro-inflammatory bacteria *Duncaniella*. In vitro and in vivo experiments confirmed that the enzymatic hydrolysis products of *Auricularia auricula* have both strong antioxidant activity and immune regulation functions. Their mechanism of action may be related to regulating cytokine networks and remodeling intestinal microbiota homeostasis to exert immune-enhancing effects, providing a theoretical basis and technical support for the high-value utilization of *Auricularia auricula* resources and the development of functional foods.

## Figures and Tables

**Table 1:** Types of proteases.

Number	Name	Manufacturer
Enzyme A	Alkaline protease	Angel Enzyme Preparation (Yichang) Co., Ltd. (Yichang, China)
Enzyme B	Trypsin	Xiasheng (Beijing) Biotechnology Development Co., Ltd. (Beijing, China)
Enzyme C	Chymotrypsin	Shanghai Yuanye Biotechnology Co., Ltd. (Shanghai, China)
Enzyme D	pepsin	
Enzyme E	Prolyl endopeptidase	Provided by Northeast Agricultural University

**Table 2:** Orthogonal experiment factors and levels for extract peptides.

Level	Factor		
	A Vitality Ratio (U:U)	B Dosage (U/g)	C Time (min)
1	1:1	5000	90
2	1.5:1	6000	120
3	2:1	7000	150

**Table 3:** Types of enzymes.

Number	Name	Manufacturer
Enzyme 1	Pectinase	Nanning Dongheng Huadao Biotechnology Co., Ltd. (Nanning, China)
Enzyme 3	Cellulase	
Enzyme 4	β—glucanase	Angel Enzyme Preparation (Yichang) Co., Ltd. (Yichang, China)
Enzyme 5	Pectinase	
Enzyme 6	Glucoamylase	
Enzyme 7	α-amylase	Xiasheng (Beijing) Biotechnology Development Co., Ltd. (Beeijing, China)
Enzyme 8	Xylanase	
Enzyme 9	β—glucanase	
Enzyme 10	Pectinase	
Enzyme 11	Glucoamylase	

**Table 4:** Orthogonal experiment factors and levels for extracting polysaccharides..

Level	Factor		
	A Time (min)	B Dosage (U/g)	C Vitality Ratio (U:U)
1	60	5000	1:2
2	90	10,000	1:1.5
3	120	15,000	1:1

**Table 5:** KM mice experimental groups and administration.

Group	Experimental Cycle		
	1~7 d	8~10 d (3 d, Intraperitoneal Injection Modeling)	11–24 d (14 d)
Con	Natural lighting, free eating and drinking	0.9% normal saline	0.9% normal saline
Mod		80 mg/kg CTX	0.9% normal saline
PC		80 mg/kg CTX	80 mg/kg Levamisole hydrochloride
AAPP1		80 mg/kg CTX	200 mg/kg AAPP1
AAPS3		80 mg/kg CTX	200 mg/kg AAPS3

**Table 6:** Orthogonal experimental results for extract peptides.

Test Number	Factor				DPPH Radical Scavenging Rate (%)
	A Vitality Ratio (U:U)	B Dosage (U/g)	C Time (min)	D (Empty Column)	
1	1	1	1	1	53.88
2	1	2	3	2	67.36
3	1	3	2	3	69.17
4	2	1	3	3	60.42
5	2	2	2	1	78.52
6	2	3	1	2	59.72
7	3	1	2	2	52.12
8	3	2	1	3	54.16
9	3	3	3	1	48.76
K1	190.41	166.42	167.76	181.16	
K2	198.66	200.04	199.81	179.20	
K3	155.04	177.65	176.54	183.75	
k1	63.47	55.47	55.92	60.39	
k2	66.22	66.68	66.60	59.73	
k3	51.68	59.22	58.85	61.25	
R	14.54	11.21	10.68	1.52	

**Table 7:** The variance analysis results for extract peptides.

Factor	Sum of Squares	Freedom	Mean Square	F Value	Significance
A	357.98	2	178.99	103.09	*
B	195.30	2	97.65	56.24	*
C	182.86	2	91.43	52.66	*
error	3.47	2	1.74		

Note: * indicates a significant effect on the results, *p* < 0.05.

**Table 8:** Orthogonal experimental results for extracting polysaccharides.

Test Number	Factor				DPPH Radical Scavenging Rate (%)
	A Time (min)	B Dosage (U/g)	C Vitality Ratio (U:U)	D (Empty Column)	
1	1	1	1	1	38.16
2	1	2	3	2	36.66
3	1	3	2	3	33.72
4	2	1	3	3	52.49
5	2	2	2	1	60.35
6	2	3	1	2	59.30
7	3	1	2	2	52.12
8	3	2	1	3	61.49
9	3	3	3	1	48.76
K1	108.54	142.77	158.95	147.27	
K2	172.14	158.50	146.19	148.08	
K2	162.37	141.78	137.91	147.70	
k1	36.18	47.59	52.98	49.09	
k2	57.38	52.83	48.73	49.36	
k3	54.12	47.26	45.97	49.23	
R	21.20	5.57	7.01	0.27	

**Table 9:** The variance analysis results for extracting polysaccharides.

Factor	Sum of Squares	Freedom	Mean Square	F Value	Significance
A	782.01	2	391.00	7142.36	*
B	58.66	2	29.33	535.79	*
C	74.90	2	37.45	684.04	*
error	3.47	2	1.74		

Note: * indicates a significant effect on the results, *p* < 0.05.

**Table 10:** Comparison of antioxidant activity of different components.

	IC50 for DPPH Scavenging (mg/mL)	IC50 for OH Scavenging (mg/mL)	IC50 for O_2_^−^ Scavenging (mg/mL)
AAP	1.88 ± 0.03	2.33 ± 0.07	2.28 ± 0.02
AAPP1	0.63 ± 0.02	1.25 ± 0.01	1.96 ± 0.07
AAPS1	0.97 ± 0.03	1.83 ± 0.06	4.26 ± 0.03
AAPS2	1.22 ± 0.07	2.73 ± 0.10	4.65 ± 0.15
AAPS3	0.53 ± 0.00	1.42 ± 0.00	2.30 ± 0.00

## Data Availability

The original contributions presented in this study are included in the article. Further inquiries can be directed to the corresponding authors.
